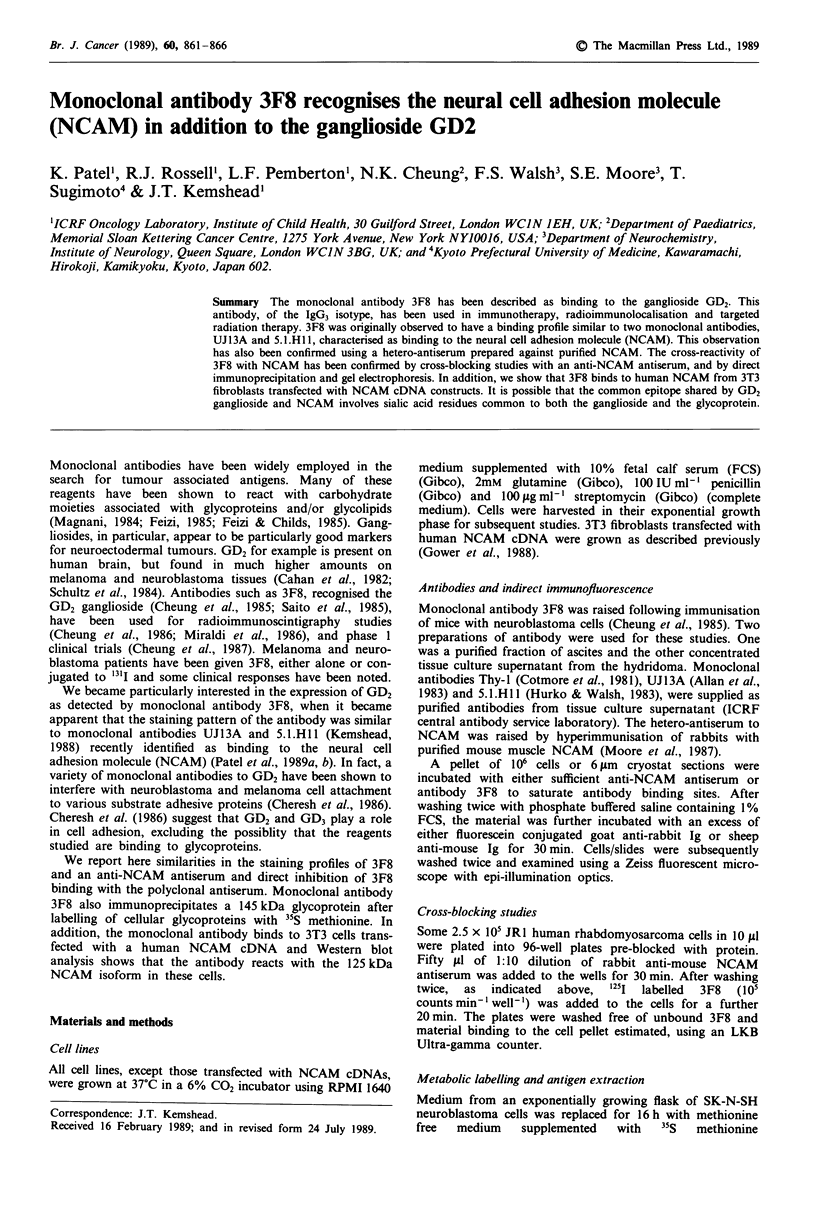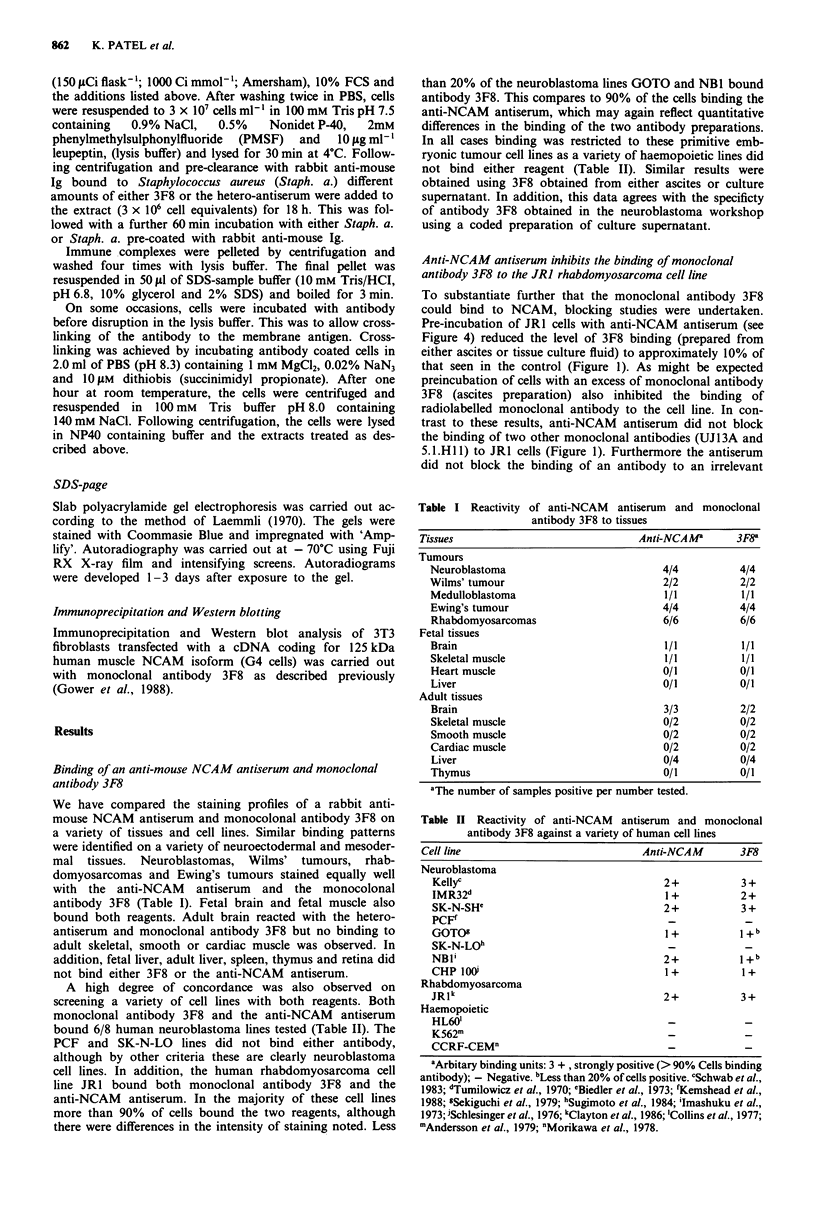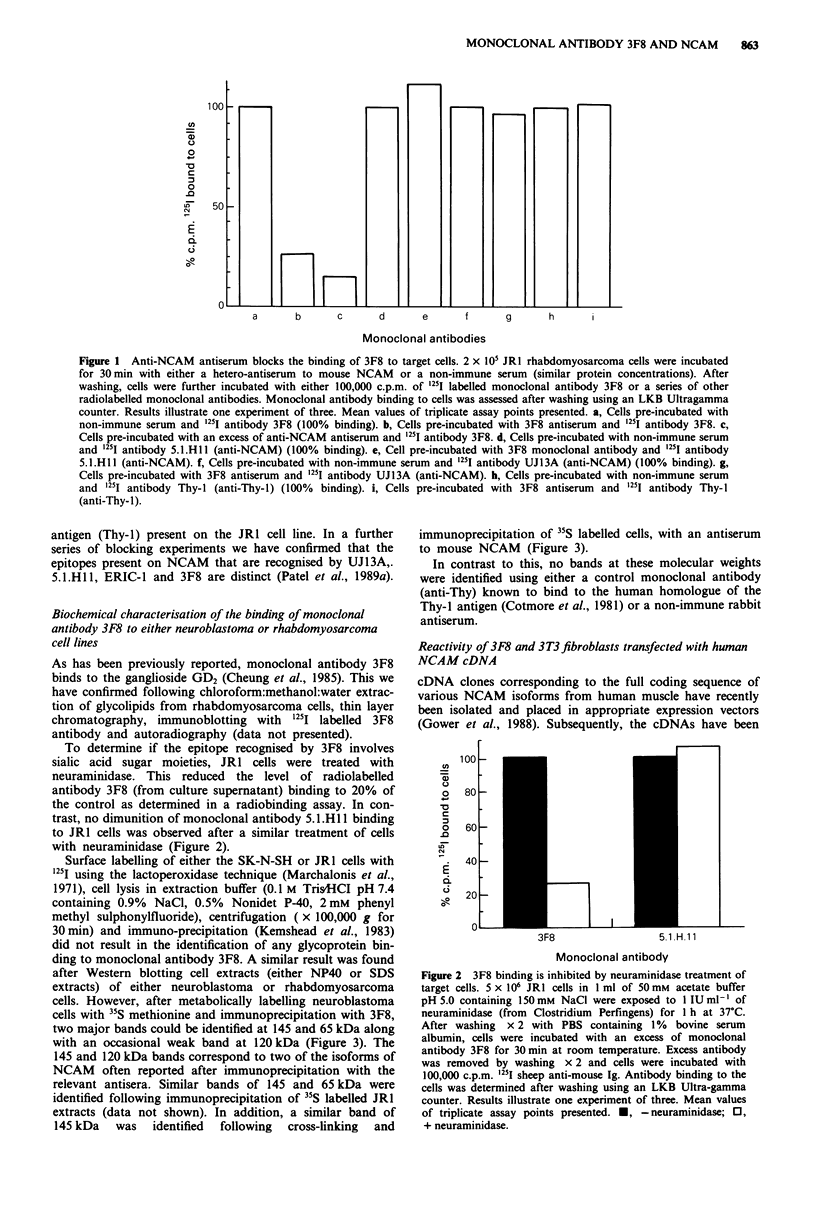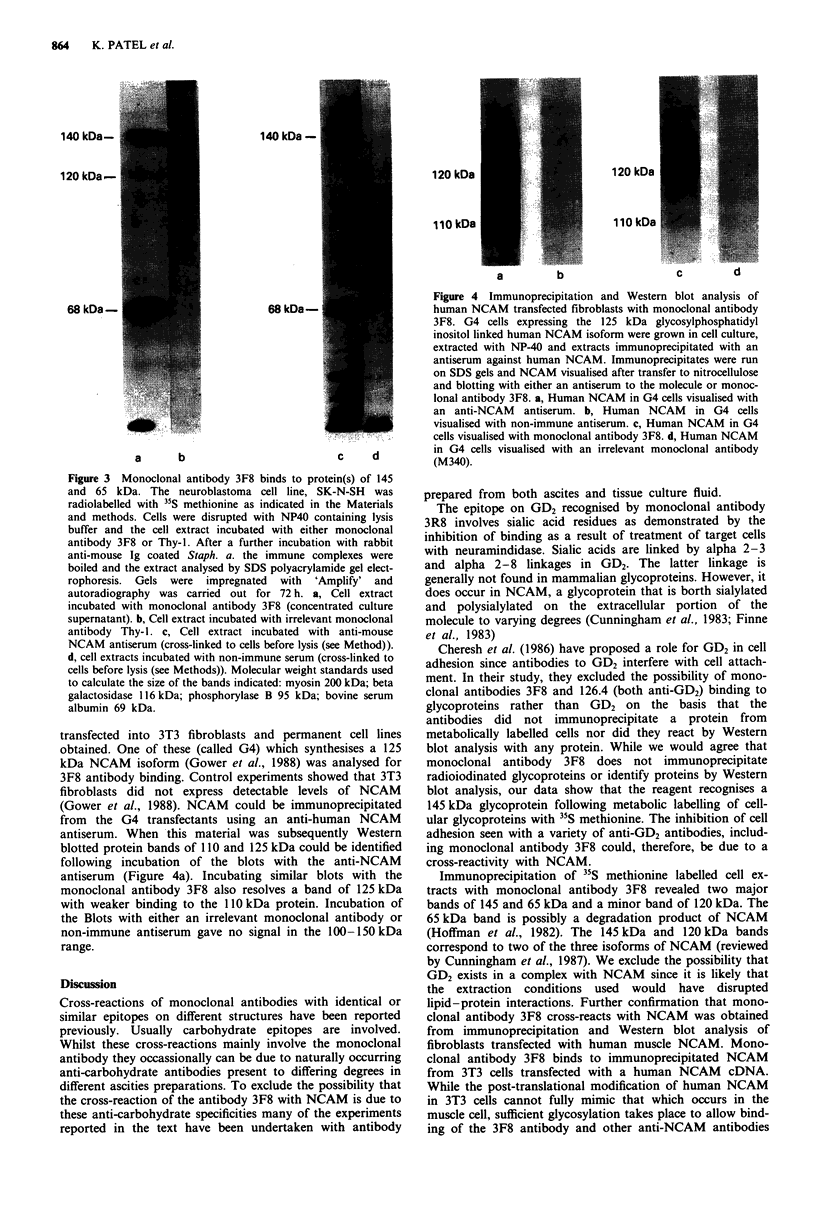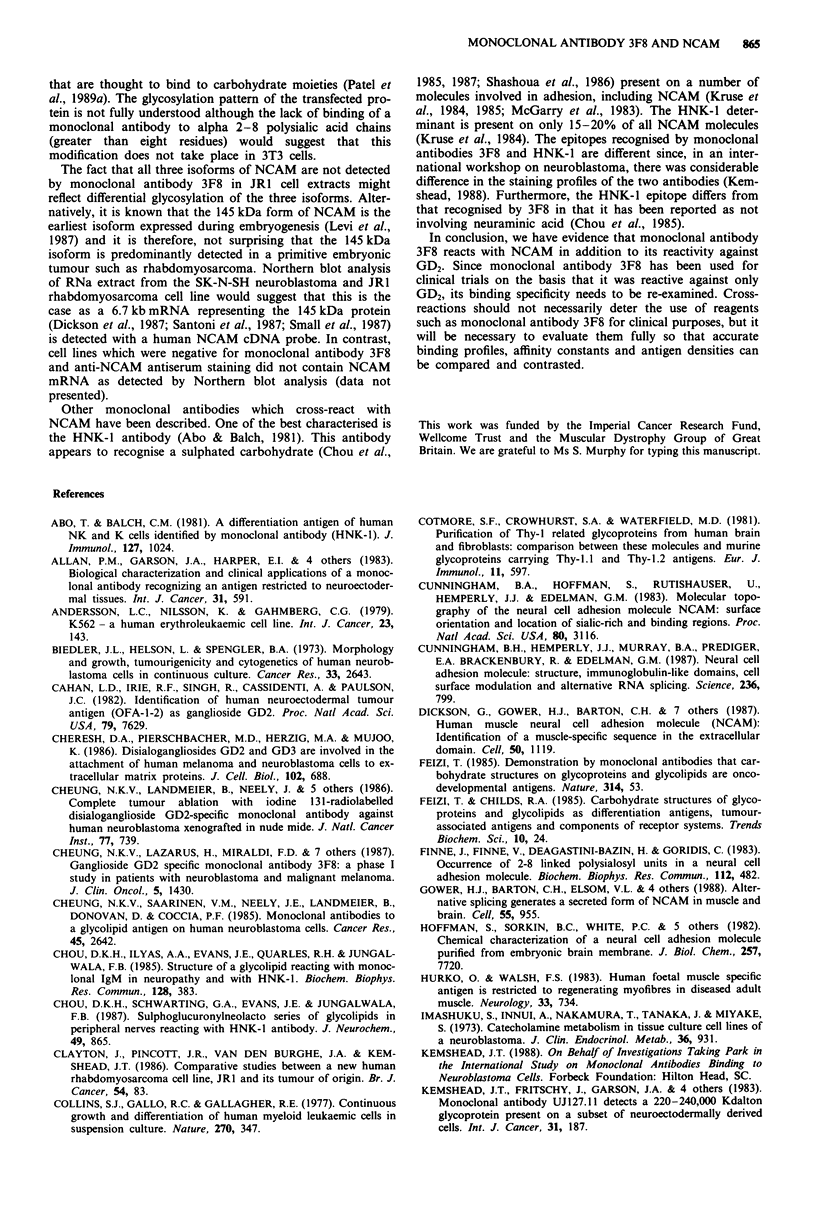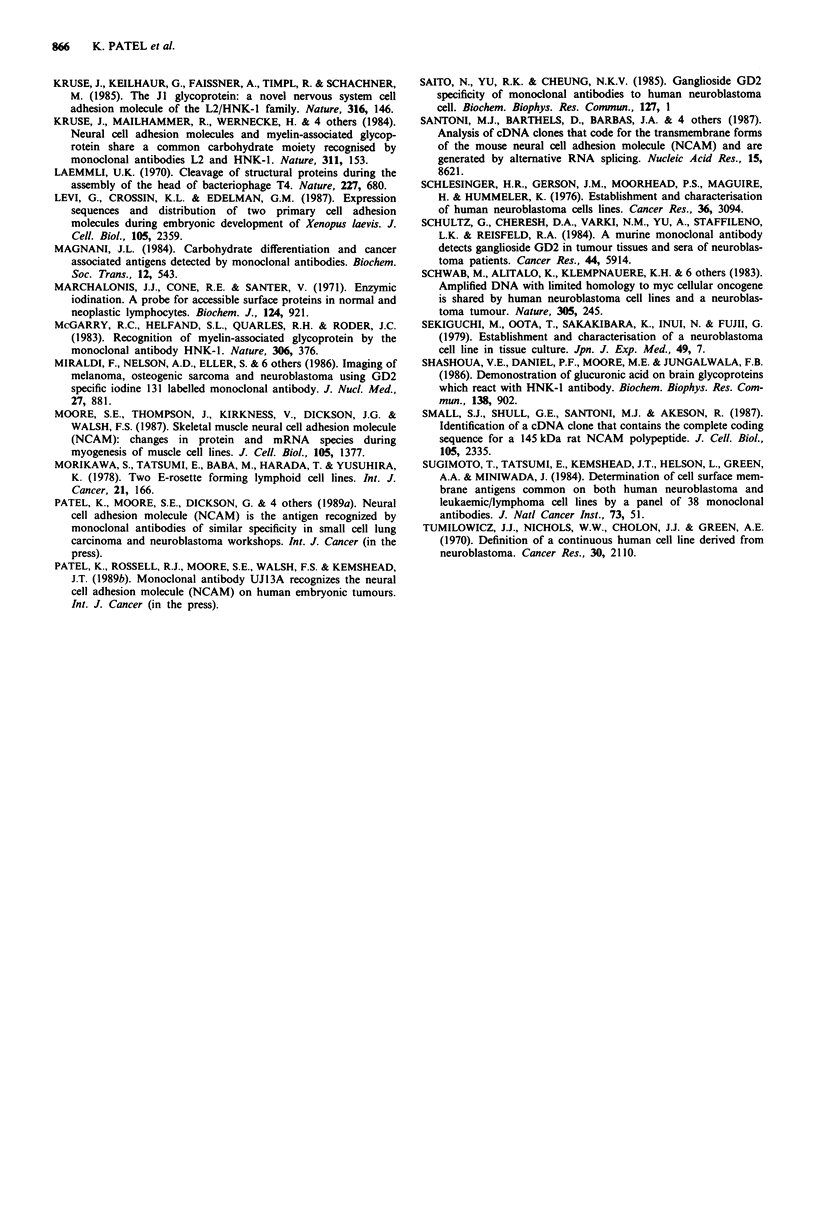# Monoclonal antibody 3F8 recognises the neural cell adhesion molecule (NCAM) in addition to the ganglioside GD2.

**DOI:** 10.1038/bjc.1989.380

**Published:** 1989-12

**Authors:** K. Patel, R. J. Rossell, L. F. Pemberton, N. K. Cheung, F. S. Walsh, S. E. Moore, T. Sugimoto, J. T. Kemshead

**Affiliations:** ICRF Oncology Laboratory, Institute of Child Health, London, UK.

## Abstract

**Images:**


Br. J. Cancer (1989), 60, 861-866 © The Macmillan Press Ltd., 1989

### Monoclonal antibody 3F8 recognises the neural cell adhesion molecule (NCAM) in addition to the ganglioside GD2

K. Patel[1], R.J. Rossell[1], L.F. Pemberton[1], N.K. Cheung[2], F.S. Walsh[3], S.E. Moore[3], T. Sugimoto[4] & J.T. Kemshead[1]

[1]*ICRF Oncology Laboratory, Institute of Child Health, 30 Guilford Street, London WC1N 1EH, UK;* [2]*Department of Paediatrics, Memorial Sloan Kettering Cancer Centre, 1275 York Avenue, New York NY10016, USA;* [3]*Department of Neurochemistry, Institute of Neurology, Queen Square, London WC1N 3BG, UK; and* [4]*Kyoto Prefectural University of Medicine, Kawaramachi, Hirokoji, Kamikyoku, Kyoto, Japan 602.*

**Summary** The monoclonal antibody 3F8 has been described as binding to the ganglioside $GD_2$. This antibody, of the $IgG_3$ isotype, has been used in immunotherapy, radioimmunolocalisation and targeted radiation therapy. 3F8 was originally observed to have a binding profile similar to two monoclonal antibodies, UJ13A and 5.1.H11, characterised as binding to the neural cell adhesion molecule (NCAM). This observation has also been confirmed using a hetero-antiserum prepared against purified NCAM. The cross-reactivity of 3F8 with NCAM has been confirmed by cross-blocking studies with an anti-NCAM antiserum, and by direct immunoprecipitation and gel electrophoresis. In addition, we show that 3F8 binds to human NCAM from 3T3 fibroblasts transfected with NCAM cDNA constructs. It is possible that the common epitope shared by $GD_2$ ganglioside and NCAM involves sialic acid residues common to both the ganglioside and the glycoprotein.

Monoclonal antibodies have been widely employed in the search for tumour associated antigens. Many of these reagents have been shown to react with carbohydrate moieties associated with glycoproteins and/or glycolipids (Magnani, 1984; Feizi, 1985; Feizi & Childs, 1985). Gangliosides, in particular, appear to be particularly good markers for neuroectodermal tumours. $GD_2$ for example is present on human brain, but found in much higher amounts on melanoma and neuroblastoma tissues (Cahan *et al.*, 1982; Schultz *et al.*, 1984). Antibodies such as 3F8, recognised the $GD_2$ ganglioside (Cheung *et al.*, 1985; Saito *et al.*, 1985), have been used for radioimmunoscintigraphy studies (Cheung *et al.*, 1986; Miraldi *et al.*, 1986), and phase 1 clinical trials (Cheung *et al.*, 1987). Melanoma and neuroblastoma patients have been given 3F8, either alone or conjugated to $^{131}I$ and some clinical responses have been noted.

We became particularly interested in the expression of $GD_2$ as detected by monoclonal antibody 3F8, when it became apparent that the staining pattern of the antibody was similar to monoclonal antibodies UJ13A and 5.1.H11 (Kemshead, 1988) recently identified as binding to the neural cell adhesion molecule (NCAM) (Patel *et al.*, 1989*a*, *b*). In fact, a variety of monoclonal antibodies to $GD_2$ have been shown to interfere with neuroblastoma and melanoma cell attachment to various substrate adhesive proteins (Cheresh *et al.*, 1986). Cheresh *et al.* (1986) suggest that $GD_2$ and $GD_3$ play a role in cell adhesion, excluding the possiblity that the reagents studied are binding to glycoproteins.

We report here similarities in the staining profiles of 3F8 and an anti-NCAM antiserum and direct inhibition of 3F8 binding with the polyclonal antiserum. Monoclonal antibody 3F8 also immunoprecipitates a 145 kDa glycoprotein after labelling of cellular glycoproteins with $^{35}S$ methionine. In addition, the monoclonal antibody binds to 3T3 cells transfected with a human NCAM cDNA and Western blot analysis shows that the antibody reacts with the 125 kDa NCAM isoform in these cells.

### Materials and methods

### *Cell lines*

All cell lines, except those transfected with NCAM cDNAs, were grown at 37°C in a 6% $CO_2$ incubator using RPMI 1640 medium supplemented with 10% fetal calf serum (FCS) (Gibco), 2mM glutamine (Gibco), 100 IU $ml^{-1}$ penicillin (Gibco) and 100 $\mu g\ ml^{-1}$ streptomycin (Gibco) (complete medium). Cells were harvested in their exponential growth phase for subsequent studies. 3T3 fibroblasts transfected with human NCAM cDNA were grown as described previously (Gower *et al.*, 1988).

### *Antibodies and indirect immunofluorescence*

Monoclonal antibody 3F8 was raised following immunisation of mice with neuroblastoma cells (Cheung *et al.*, 1985). Two preparations of antibody were used for these studies. One was a purified fraction of ascites and the other concentrated tissue culture supernatant from the hydridoma. Monoclonal antibodies Thy-1 (Cotmore *et al.*, 1981), UJ13A (Allan *et al.*, 1983) and 5.1.H11 (Hurko & Walsh, 1983), were supplied as purified antibodies from tissue culture supernatant (ICRF central antibody service laboratory). The hetero-antiserum to NCAM was raised by hyperimmunisation of rabbits with purified mouse muscle NCAM (Moore *et al.*, 1987).

A pellet of $10^6$ cells or 6 $\mu$m cryostat sections were incubated with either sufficient anti-NCAM antiserum or antibody 3F8 to saturate antibody binding sites. After washing twice with phosphate buffered saline containing 1% FCS, the material was further incubated with an excess of either fluorescein conjugated goat anti-rabbit Ig or sheep anti-mouse Ig for 30 min. Cells/slides were subsequently washed twice and examined using a Zeiss fluorescent microscope with epi-illumination optics.

### *Cross-blocking studies*

Some $2.5 \times 10^5$ JR1 human rhabdomyosarcoma cells in 10 $\mu$l were plated into 96-well plates pre-blocked with protein. Fifty $\mu$l of 1:10 dilution of rabbit anti-mouse NCAM antiserum was added to the wells for 30 min. After washing twice, as indicated above, $^{125}I$ labelled 3F8 ($10^5$ counts $min^{-1}$ $well^{-1}$) was added to the cells for a further 20 min. The plates were washed free of unbound 3F8 and material binding to the cell pellet estimated, using an LKB Ultra-gamma counter.

### *Metabolic labelling and antigen extraction*

Medium from an exponentially growing flask of SK-N-SH neuroblastoma cells was replaced for 16 h with methionine free medium supplemented with $^{35}S$ methionine

Correspondence: J.T. Kemshead.
Received 16 February 1989; and in revised form 24 July 1989.

(150 μCi $flask^{-1}$; 1000 Ci $mmol^{-1}$; Amersham), 10% FCS and the additions listed above. After washing twice in PBS, cells were resuspended to $3 \times 10^7$ cells $ml^{-1}$ in 100 mM Tris pH 7.5 containing 0.9% NaCl, 0.5% Nonidet P-40, 2mM phenylmethylsulphonylfluoride (PMSF) and 10 μg $ml^{-1}$ leupeptin, (lysis buffer) and lysed for 30 min at 4°C. Following centrifugation and pre-clearance with rabbit anti-mouse Ig bound to *Staphylococcus aureus* (*Staph. a.*) different amounts of either 3F8 or the hetero-antiserum were added to the extract ($3 \times 10^6$ cell equivalents) for 18 h. This was followed with a further 60 min incubation with either *Staph. a.* or *Staph. a.* pre-coated with rabbit anti-mouse Ig.

Immune complexes were pelleted by centrifugation and washed four times with lysis buffer. The final pellet was resuspended in 50 μl of SDS-sample buffer (10 mM Tris/HCI, pH 6.8, 10% glycerol and 2% SDS) and boiled for 3 min.

On some occasions, cells were incubated with antibody before disruption in the lysis buffer. This was to allow cross-linking of the antibody to the membrane antigen. Cross-linking was achieved by incubating antibody coated cells in 2.0 ml of PBS (pH 8.3) containing 1 mM $MgCl_2$, 0.02% $NaN_3$ and 10 μM dithiobis (succinimidyl propionate). After one hour at room temperature, the cells were centrifuged and resuspended in 100 mM Tris buffer pH 8.0 containing 140 mM NaCl. Following centrifugation, the cells were lysed in NP40 containing buffer and the extracts treated as described above.

### *SDS-page*

Slab polyacrylamide gel electrophoresis was carried out according to the method of Laemmli (1970). The gels were stained with Coommasie Blue and impregnated with 'Amplify'. Autoradiography was carried out at − 70°C using Fuji RX X-ray film and intensifying screens. Autoradiograms were developed 1–3 days after exposure to the gel.

### *Immunoprecipitation and Western blotting*

Immunoprecipitation and Western blot analysis of 3T3 fibroblasts transfected with a cDNA coding for 125 kDa human muscle NCAM isoform (G4 cells) was carried out with monoclonal antibody 3F8 as described previously (Gower et al., 1988).

### Results

### *Binding of an anti-mouse NCAM antiserum and monoclonal antibody 3F8*

We have compared the staining profiles of a rabbit anti-mouse NCAM antiserum and monocolonal antibody 3F8 on a variety of tissues and cell lines. Similar binding patterns were identified on a variety of neuroectodermal and mesodermal tissues. Neuroblastomas, Wilms' tumours, rhabdomyosarcomas and Ewing's tumours stained equally well with the anti-NCAM antiserum and the monocolonal antibody 3F8 (Table I). Fetal brain and fetal muscle also bound both reagents. Adult brain reacted with the hetero-antiserum and monoclonal antibody 3F8 but no binding to adult skeletal, smooth or cardiac muscle was observed. In addition, fetal liver, adult liver, spleen, thymus and retina did not bind either 3F8 or the anti-NCAM antiserum.

A high degree of concordance was also observed on screening a variety of cell lines with both reagents. Both monoclonal antibody 3F8 and the anti-NCAM antiserum bound 6/8 human neuroblastoma lines tested (Table II). The PCF and SK-N-LO lines did not bind either antibody, although by other criteria these are clearly neuroblastoma cell lines. In addition, the human rhabdomyosarcoma cell line JR1 bound both monoclonal antibody 3F8 and the anti-NCAM antiserum. In the majority of these cell lines more than 90% of cells bound the two reagents, although there were differences in the intensity of staining noted. Less than 20% of the neuroblastoma lines GOTO and NB1 bound antibody 3F8. This compares to 90% of the cells binding the anti-NCAM antiserum, which may again reflect quantitative differences in the binding of the two antibody preparations. In all cases binding was restricted to these primitive embryonic tumour cell lines as a variety of haemopoietic lines did not bind either reagent (Table II). Similar results were obtained using 3F8 obtained from either ascites or culture supernatant. In addition, this data agrees with the specificty of antibody 3F8 obtained in the neuroblastoma workshop using a coded preparation of culture supernatant.

### *Anti-NCAM antiserum inhibits the binding of monoclonal antibody 3F8 to the JR1 rhabdomyosarcoma cell line*

To substantiate further that the monoclonal antibody 3F8 could bind to NCAM, blocking studies were undertaken. Pre-incubation of JR1 cells with anti-NCAM antiserum (see Figure 4) reduced the level of 3F8 binding (prepared from either ascites or tissue culture fluid) to approximately 10% of that seen in the control (Figure 1). As might be expected preincubation of cells with an excess of monoclonal antibody 3F8 (ascites preparation) also inhibited the binding of radiolabelled monoclonal antibody to the cell line. In contrast to these results, anti-NCAM antiserum did not block the binding of two other monoclonal antibodies (UJ13A and 5.1.H11) to JR1 cells (Figure 1). Furthermore the antiserum did not block the binding of an antibody to an irrelevant

**Table I** Reactivity of anti-NCAM antiserum and monoclonal antibody 3F8 to tissues

| *Tissues* | *Anti-NCAM*[a] | *3F8*[a] |
|---|---|---|
| Tumours | | |
| Neuroblastoma | 4/4 | 4/4 |
| Wilms' tumour | 2/2 | 2/2 |
| Medulloblastoma | 1/1 | 1/1 |
| Ewing's tumour | 4/4 | 4/4 |
| Rhabdomyosarcomas | 6/6 | 6/6 |
| Fetal tissues | | |
| Brain | 1/1 | 1/1 |
| Skeletal muscle | 1/1 | 1/1 |
| Heart muscle | 0/1 | 0/1 |
| Liver | 0/1 | 0/1 |
| Adult tissues | | |
| Brain | 3/3 | 2/2 |
| Skeletal muscle | 0/2 | 0/2 |
| Smooth muscle | 0/2 | 0/2 |
| Cardiac muscle | 0/2 | 0/2 |
| Liver | 0/4 | 0/4 |
| Thymus | 0/1 | 0/1 |

[a]The number of samples positive per number tested.

**Table II** Reactivity of anti-NCAM antiserum and monoclonal antibody 3F8 against a variety of human cell lines

| *Cell line* | *Anti-NCAM* | *3F8* |
|---|---|---|
| Neuroblastoma | | |
| Kelly[c] | 2+ | 3+ |
| IMR32[d] | 1+ | 2+ |
| SK-N-SH[e] | 2+ | 3+ |
| PCF[f] | − | − |
| GOTO[g] | 1+ | 1+[b] |
| SK-N-LO[h] | − | − |
| NB1[i] | 2+ | 1+[b] |
| CHP 100[j] | 1+ | 1+ |
| Rhabdomyosarcoma | | |
| JR1[k] | 2+ | 3+ |
| Haemopoietic | | |
| HL60[l] | − | − |
| K562[m] | − | − |
| CCRF-CEM[n] | − | − |

[a]Arbitary binding units: 3 +, strongly positive (> 90% Cells binding antibody); − Negative. [b]Less than 20% of cells positive. [c]Schwab et al., 1983; [d]Tumilowicz et al., 1970; [e]Biedler et al., 1973; [f]Kemshead et al., 1988; [g]Sekiguchi et al., 1979; [h]Sugimoto et al., 1984; [i]Imashuku et al., 1973; [j]Schlesinger et al., 1976; [k]Clayton et al., 1986; [l]Collins et al., 1977; [m]Andersson et al., 1979; [n]Morikawa et al., 1978.

**Figure 1** Anti-NCAM antiserum blocks the binding of 3F8 to target cells. $2 \times 10^5$ JR1 rhabdomyosarcoma cells were incubated for 30 min with either a hetero-antiserum to mouse NCAM or a non-immune serum (similar protein concentrations). After washing, cells were further incubated with either 100,000 c.p.m. of $^{125}I$ labelled monoclonal antibody 3F8 or a series of other radiolabelled monoclonal antibodies. Monoclonal antibody binding to cells was assessed after washing using an LKB Ultragamma counter. Results illustrate one experiment of three. Mean values of triplicate assay points presented. **a**, Cells pre-incubated with non-immune serum and $^{125}I$ antibody 3F8 (100% binding). **b**, Cells pre-incubated with 3F8 antiserum and $^{125}I$ antibody 3F8. **c**, Cells pre-incubated with an excess of anti-NCAM antiserum and $^{125}I$ antibody 3F8. **d**, Cells pre-incubated with non-immune serum and $^{125}I$ antibody 5.1.H11 (anti-NCAM) (100% binding). **e**, Cell pre-incubated with 3F8 monoclonal antibody and $^{125}I$ antibody 5.1.H11 (anti-NCAM). **f**, Cells pre-incubated with non-immune serum and $^{125}I$ antibody UJ13A (anti-NCAM) (100% binding). **g**, Cells pre-incubated with 3F8 antiserum and $^{125}I$ antibody UJ13A (anti-NCAM). **h**, Cells pre-incubated with non-immune serum and $^{125}I$ antibody Thy-1 (anti-Thy-1) (100% binding). **i**, Cells pre-incubated with 3F8 antiserum and $^{125}I$ antibody Thy-1 (anti-Thy-1).

antigen (Thy-1) present on the JR1 cell line. In a further series of blocking experiments we have confirmed that the epitopes present on NCAM that are recognised by UJ13A,. 5.1.H11, ERIC-1 and 3F8 are distinct (Patel *et al.*, 1989*a*).

### *Biochemical characterisation of the binding of monoclonal antibody 3F8 to either neuroblastoma or rhabdomyosarcoma cell lines*

As has been previously reported, monoclonal antibody 3F8 binds to the ganglioside $GD_2$ (Cheung *et al.*, 1985). This we have confirmed following chloroform:methanol:water extraction of glycolipids from rhabdomyosarcoma cells, thin layer chromatography, immunoblotting with $^{125}I$ labelled 3F8 antibody and autoradiography (data not presented).

To determine if the epitope recognised by 3F8 involves sialic acid sugar moieties, JR1 cells were treated with neuraminidase. This reduced the level of radiolabelled antibody 3F8 (from culture supernatant) binding to 20% of the control as determined in a radiobinding assay. In contrast, no dimunition of monoclonal antibody 5.1.H11 binding to JR1 cells was observed after a similar treatment of cells with neuraminidase (Figure 2).

Surface labelling of either the SK-N-SH or JR1 cells with $^{125}I$ using the lactoperoxidase technique (Marchalonis *et al.*, 1971), cell lysis in extraction buffer (0.1 M Tris/HCl pH 7.4 containing 0.9% NaCl, 0.5% Nonidet P-40, 2 mM phenyl methyl sulphonylfluoride), centrifugation ( $\times$ 100,000 *g* for 30 min) and immuno-precipitation (Kemshead *et al.*, 1983) did not result in the identification of any glycoprotein binding to monoclonal antibody 3F8. A similar result was found after Western blotting cell extracts (either NP40 or SDS extracts) of either neuroblastoma or rhabdomyosarcoma cells. However, after metabolically labelling neuroblastoma cells with $^{35}S$ methionine and immunoprecipitation with 3F8, two major bands could be identified at 145 and 65 kDa along with an occasional weak band at 120 kDa (Figure 3). The 145 and 120 kDa bands correspond to two of the isoforms of NCAM often reported after immunoprecipitation with the relevant antisera. Similar bands of 145 and 65 kDa were identified following immunoprecipitation of $^{35}S$ labelled JR1 extracts (data not shown). In addition, a similar band of 145 kDa was identified following cross-linking and immunoprecipitation of $^{35}S$ labelled cells, with an antiserum to mouse NCAM (Figure 3).

In contrast to this, no bands at these molecular weights were identified using either a control monoclonal antibody (anti-Thy) known to bind to the human homologue of the Thy-1 antigen (Cotmore *et al.*, 1981) or a non-immune rabbit antiserum.

### *Reactivity of 3F8 and 3T3 fibroblasts transfected with human NCAM cDNA*

cDNA clones corresponding to the full coding sequence of various NCAM isoforms from human muscle have recently been isolated and placed in appropriate expression vectors (Gower *et al.*, 1988). Subsequently, the cDNAs have been

**Figure 2** 3F8 binding is inhibited by neuraminidase treatment of target cells. $5 \times 10^6$ JR1 cells in 1 ml of 50 mM acetate buffer pH 5.0 containing 150 mM NaCl were exposed to 1 IU $ml^{-1}$ of neuraminidase (from Clostridium Perfingens) for 1 h at 37°C. After washing $\times$ 2 with PBS containing 1% bovine serum albumin, cells were incubated with an excess of monoclonal antibody 3F8 for 30 min at room temperature. Excess antibody was removed by washing $\times$ 2 and cells were incubated with 100,000 c.p.m. $^{125}I$ sheep anti-mouse Ig. Antibody binding to the cells was determined after washing using an LKB Ultra-gamma counter. Results illustrate one experiment of three. Mean values of triplicate assay points presented. ■, − neuraminidase; □, + neuraminidase.

**Figure 3** Monoclonal antibody 3F8 binds to protein(s) of 145 and 65 kDa. The neuroblastoma cell line, SK-N-SH was radiolabelled with $^{35}S$ methionine as indicated in the Materials and methods. Cells were disrupted with NP40 containing lysis buffer and the cell extract incubated with either monoclonal antibody 3F8 or Thy-1. After a further incubation with rabbit anti-mouse Ig coated *Staph. a.* the immune complexes were boiled and the extract analysed by SDS polyacrylamide gel electrophoresis. Gels were impregnated with 'Amplify' and autoradiography was carried out for 72 h. **a**, Cell extract incubated with monoclonal antibody 3F8 (concentrated culture supernatant). **b**, Cell extract incubated with irrelevant monoclonal antibody Thy-1. **c**, Cell extract incubated with anti-mouse NCAM antiserum (cross-linked to cells before lysis (see Method)). **d**, cell extracts incubated with non-immune serum (cross-linked to cells before lysis (see Methods)). Molecular weight standards used to calculate the size of the bands indicated: myosin 200 kDa; beta galactosidase 116 kDa; phosphorylase B 95 kDa; bovine serum albumin 69 kDa.

transfected into 3T3 fibroblasts and permanent cell lines obtained. One of these (called G4) which synthesises a 125 kDa NCAM isoform (Gower *et al.*, 1988) was analysed for 3F8 antibody binding. Control experiments showed that 3T3 fibroblasts did not express detectable levels of NCAM (Gower *et al.*, 1988). NCAM could be immunoprecipitated from the G4 transfectants using an anti-human NCAM antiserum. When this material was subsequently Western blotted protein bands of 110 and 125 kDa could be identified following incubation of the blots with the anti-NCAM antiserum (Figure 4a). Incubating similar blots with the monoclonal antibody 3F8 also resolves a band of 125 kDa with weaker binding to the 110 kDa protein. Incubation of the Blots with either an irrelevant monoclonal antibody or non-immune antiserum gave no signal in the 100–150 kDa range.

### Discussion

Cross-reactions of monoclonal antibodies with identical or similar epitopes on different structures have been reported previously. Usually carbohydrate epitopes are involved. Whilst these cross-reactions mainly involve the monoclonal antibody they occassionally can be due to naturally occurring anti-carbohydrate antibodies present to differing degrees in different ascities preparations. To exclude the possibility that the cross-reaction of the antibody 3F8 with NCAM is due to these anti-carbohydrate specificities many of the experiments reported in the text have been undertaken with antibody

**Figure 4** Immunoprecipitation and Western blot analysis of human NCAM transfected fibroblasts with monoclonal antibody 3F8. G4 cells expressing the 125 kDa glycosylphosphatidyl inositol linked human NCAM isoform were grown in cell culture, extracted with NP-40 and extracts immunoprecipitated with an antiserum against human NCAM. Immunoprecipitates were run on SDS gels and NCAM visualised after transfer to nitrocellulose and blotting with either an antiserum to the molecule or monoclonal antibody 3F8. **a**, Human NCAM in G4 cells visualised with an anti-NCAM antiserum. **b**, Human NCAM in G4 cells visualised with non-immune antiserum. **c**, Human NCAM in G4 cells visualised with monoclonal antibody 3F8. **d**, Human NCAM in G4 cells visualised with an irrelevant monoclonal antibody (M340).

prepared from both ascites and tissue culture fluid.

The epitope on $GD_2$ recognised by monoclonal antibody 3R8 involves sialic acid residues as demonstrated by the inhibition of binding as a result of treatment of target cells with neuramindidase. Sialic acids are linked by alpha 2–3 and alpha 2–8 linkages in $GD_2$. The latter linkage is generally not found in mammalian glycoproteins. However, it does occur in NCAM, a glycoprotein that is borth sialylated and polysialylated on the extracellular portion of the molecule to varying degrees (Cunningham *et al.*, 1983; Finne *et al.*, 1983)

Cheresh *et al.* (1986) have proposed a role for $GD_2$ in cell adhesion since antibodies to $GD_2$ interfere with cell attachment. In their study, they excluded the possibility of monoclonal antibodies 3F8 and 126.4 (both anti-$GD_2$) binding to glycoproteins rather than $GD_2$ on the basis that the antibodies did not immunoprecipitate a protein from metabolically labelled cells nor did they react by Western blot analysis with any protein. While we would agree that monoclonal antibody 3F8 does not immunoprecipitate radioiodinated glycoproteins or identify proteins by Western blot analysis, our data show that the reagent recognises a 145 kDa glycoprotein following metabolic labelling of cellular glycoproteins with $^{35}S$ methionine. The inhibition of cell adhesion seen with a variety of anti-$GD_2$ antibodies, including monoclonal antibody 3F8 could, therefore, be due to a cross-reactivity with NCAM.

Immunoprecipitation of $^{35}S$ methionine labelled cell extracts with monoclonal antibody 3F8 revealed two major bands of 145 and 65 kDa and a minor band of 120 kDa. The 65 kDa band is possibly a degradation product of NCAM (Hoffman *et al.*, 1982). The 145 kDa and 120 kDa bands correspond to two of the three isoforms of NCAM (reviewed by Cunningham *et al.*, 1987). We exclude the possibility that $GD_2$ exists in a complex with NCAM since it is likely that the extraction conditions used would have disrupted lipid–protein interactions. Further confirmation that monoclonal antibody 3F8 cross-reacts with NCAM was obtained from immunoprecipitation and Western blot analysis of fibroblasts transfected with human muscle NCAM. Monoclonal antibody 3F8 binds to immunoprecipitated NCAM from 3T3 cells transfected with a human NCAM cDNA. While the post-translational modification of human NCAM in 3T3 cells cannot fully mimic that which occurs in the muscle cell, sufficient glycosylation takes place to allow binding of the 3F8 antibody and other anti-NCAM antibodies that are thought to bind to carbohydrate moieties (Patel *et al.*, 1989*a*). The glycosylation pattern of the transfected protein is not fully understood although the lack of binding of a monoclonal antibody to alpha 2–8 polysialic acid chains (greater than eight residues) would suggest that this modification does not take place in 3T3 cells.

The fact that all three isoforms of NCAM are not detected by monoclonal antibody 3F8 in JR1 cell extracts might reflect differential glycosylation of the three isoforms. Alternatively, it is known that the 145 kDa form of NCAM is the earliest isoform expressed during embryogenesis (Levi *et al.*, 1987) and it is therefore, not surprising that the 145 kDa isoform is predominantly detected in a primitive embryonic tumour such as rhabdomyosarcoma. Northern blot analysis of RNa extract from the SK-N-SH neuroblastoma and JR1 rhabdomyosarcoma cell line would suggest that this is the case as a 6.7 kb mRNA representing the 145 kDa protein (Dickson *et al.*, 1987; Santoni *et al.*, 1987; Small *et al.*, 1987) is detected with a human NCAM cDNA probe. In contrast, cell lines which were negative for monoclonal antibody 3F8 and anti-NCAM antiserum staining did not contain NCAM mRNA as detected by Northern blot analysis (data not presented).

Other monoclonal antibodies which cross-react with NCAM have been described. One of the best characterised is the HNK-1 antibody (Abo & Balch, 1981). This antibody appears to recognise a sulphated carbohydrate (Chou *et al.*, 1985, 1987; Shashoua *et al.*, 1986) present on a number of molecules involved in adhesion, including NCAM (Kruse *et al.*, 1984, 1985; McGarry *et al.*, 1983). The HNK-1 determinant is present on only 15–20% of all NCAM molecules (Kruse *et al.*, 1984). The epitopes recognised by monoclonal antibodies 3F8 and HNK-1 are different since, in an international workshop on neuroblastoma, there was considerable difference in the staining profiles of the two antibodies (Kemshead, 1988). Furthermore, the HNK-1 epitope differs from that recognised by 3F8 in that it has been reported as not involving neuraminic acid (Chou *et al.*, 1985).

In conclusion, we have evidence that monoclonal antibody 3F8 reacts with NCAM in addition to its reactivity against $GD_2$. Since monoclonal antibody 3F8 has been used for clinical trials on the basis that it was reactive against only $GD_2$, its binding specificity needs to be re-examined. Cross-reactions should not necessarily deter the use of reagents such as monoclonal antibody 3F8 for clinical purposes, but it will be necessary to evaluate them fully so that accurate binding profiles, affinity constants and antigen densities can be compared and contrasted.

This work was funded by the Imperial Cancer Research Fund, Wellcome Trust and the Muscular Dystrophy Group of Great Britain. We are grateful to Ms S. Murphy for typing this manuscript.

### References

ABO, T. & BALCH, C.M. (1981). A differentiation antigen of human NK and K cells identified by monoclonal antibody (HNK-1). *J. Immunol.*, **127**, 1024.

ALLAN, P.M., GARSON, J.A., HARPER, E.I. & 4 others (1983). Biological characterization and clinical applications of a monoclonal antibody recognizing an antigen restricted to neuroectodermal tissues. *Int. J. Cancer*, **31**, 591.

ANDERSSON, L.C., NILSSON, K. & GAHMBERG, C.G. (1979). K562 – a human erythroleukaemic cell line. *Int. J. Cancer*, **23**, 143.

BIEDLER, J.L., HELSON, L. & SPENGLER, B.A. (1973). Morphology and growth, tumourigenicity and cytogenetics of human neuroblastoma cells in continuous culture. *Cancer Res.*, **33**, 2643.

CAHAN, L.D., IRIE, R.F., SINGH, R., CASSIDENTI, A. & PAULSON, J.C. (1982). Identification of human neuroectodermal tumour antigen (OFA-1-2) as ganglioside GD2. *Proc. Natl Acad. Sci. USA*, **79**, 7629.

CHERESH, D.A., PIERSCHBACHER, M.D., HERZIG, M.A. & MUJOO, K. (1986). Disialogangliosides GD2 and GD3 are involved in the attachment of human melanoma and neuroblastoma cells to extracellular matrix proteins. *J. Cell. Biol.*, **102**, 688.

CHEUNG, N.K.V., LANDMEIER, B., NEELY, J. & 5 others (1986). Complete tumour ablation with iodine 131-radiolabelled disialoganglioside GD2-specific monoclonal antibody against human neuroblastoma xenografted in nude mide. *J. Natl. Cancer Inst.*, **77**, 739.

CHEUNG, N.K.V., LAZARUS, H., MIRALDI, F.D. & 7 others (1987). Ganglioside GD2 specific monoclonal antibody 3F8: a phase I study in patients with neuroblastoma and malignant melanoma. *J. Clin. Oncol.*, **5**, 1430.

CHEUNG, N.K.V., SAARINEN, V.M., NEELY, J.E., LANDMEIER, B., DONOVAN, D. & COCCIA, P.F. (1985). Monoclonal antibodies to a glycolipid antigen on human neuroblastoma cells. *Cancer Res.*, **45**, 2642.

CHOU, D.K.H., ILYAS, A.A., EVANS, J.E., QUARLES, R.H. & JUNGALWALA, F.B. (1985). Structure of a glycolipid reacting with monoclonal IgM in neuropathy and with HNK-1. *Biochem. Biophys. Res. Commun.*, **128**, 383.

CHOU, D.K.H., SCHWARTING, G.A., EVANS, J.E. & JUNGALWALA, F.B. (1987). Sulphoglucuronylneolacto series of glycolipids in peripheral nerves reacting with HNK-1 antibody. *J. Neurochem.*, **49**, 865.

CLAYTON, J., PINCOTT, J.R., VAN DEN BURGHE, J.A. & KEMSHEAD, J.T. (1986). Comparative studies between a new human rhabdomyosarcoma cell line, JR1 and its tumour of origin. *Br. J. Cancer*, **54**, 83.

COLLINS, S.J., GALLO, R.C. & GALLAGHER, R.E. (1977). Continuous growth and differentiation of human myeloid leukaemic cells in suspension culture. *Nature*, **270**, 347.

COTMORE, S.F., CROWHURST, S.A. & WATERFIELD, M.D. (1981). Purification of Thy-1 related glycoproteins from human brain and fibroblasts: comparison between these molecules and murine glycoproteins carrying Thy-1.1 and Thy-1.2 antigens. *Eur. J. Immunol.*, **11**, 597.

CUNNINGHAM, B.A., HOFFMAN, S., RUTISHAUSER, U., HEMPERLY, J.J. & EDELMAN, G.M. (1983). Molecular topography of the neural cell adhesion molecule NCAM: surface orientation and location of sialic-rich and binding regions. *Proc. Natl Acad. Sci. USA*, **80**, 3116.

CUNNINGHAM, B.H., HEMPERLY, J.J., MURRAY, B.A., PREDIGER, E.A. BRACKENBURY, R. & EDELMAN, G.M. (1987). Neural cell adhesion molecule: structure, immunoglobulin-like domains, cell surface modulation and alternative RNA splicing. *Science*, **236**, 799.

DICKSON, G., GOWER, H.J., BARTON, C.H. & 7 others (1987). Human muscle neural cell adhesion molecule (NCAM): Identification of a muscle-specific sequence in the extracellular domain. *Cell*, **50**, 1119.

FEIZI, T. (1985). Demonstration by monoclonal antibodies that carbohydrate structures on glycoproteins and glycolipids are oncodevelopmental antigens. *Nature*, **314**, 53.

FEIZI, T. & CHILDS, R.A. (1985). Carbohydrate structures of glycoproteins and glycolipids as differentiation antigens, tumourassociated antigens and components of receptor systems. *Trends Biochem. Sci.*, **10**, 24.

FINNE, J., FINNE, V., DEAGASTINI-BAZIN, H. & GORIDIS, C. (1983). Occurrence of 2-8 linked polysialosyl units in a neural cell adhesion molecule. *Biochem. Biophys. Res. Commun.*, **112**, 482.

GOWER, H.J., BARTON, C.H., ELSOM, V.L. & 4 others (1988). Alternative splicing generates a secreted form of NCAM in muscle and brain. *Cell*, **55**, 955.

HOFFMAN, S., SORKIN, B.C., WHITE, P.C. & 5 others (1982). Chemical characterization of a neural cell adhesion molecule purified from embryonic brain membrane. *J. Biol. Chem.*, **257**, 7720.

HURKO, O. & WALSH, F.S. (1983). Human foetal muscle specific antigen is restricted to regenerating myofibres in diseased adult muscle. *Neurology*, **33**, 734.

IMASHUKU, S., INNUI, A., NAKAMURA, T., TANAKA, J. & MIYAKE, S. (1973). Catecholamine metabolism in tissue culture cell lines of a neuroblastoma. *J. Clin. Endocrinol. Metab.*, **36**, 931.

KEMSHEAD, J.T. (1988). *On Behalf of Investigations Taking Park in the International Study on Monoclonal Antibodies Binding to Neuroblastoma Cells.* Forbeck Foundation: Hilton Head, SC.

KEMSHEAD, J.T., FRITSCHY, J., GARSON, J.A. & 4 others (1983). Monoclonal antibody UJ127.11 detects a 220–240,000 Kdalton glycoprotein present on a subset of neuroectodermally derived cells. *Int. J. Cancer*, **31**, 187.

KRUSE, J., KEILHAUR, G., FAISSNER, A., TIMPL, R. & SCHACHNER, M. (1985). The J1 glycoprotein: a novel nervous system cell adhesion molecule of the L2/HNK-1 family. *Nature*, **316**, 146.

KRUSE, J., MAILHAMMER, R., WERNECKE, H. & 4 others (1984). Neural cell adhesion molecules and myelin-associated glycoprotein share a common carbohydrate moiety recognised by monoclonal antibodies L2 and HNK-1. *Nature*, **311**, 153.

LAEMMLI, U.K. (1970). Cleavage of structural proteins during the assembly of the head of bacteriophage T4. *Nature*, **227**, 680.

LEVI, G., CROSSIN, K.L. & EDELMAN, G.M. (1987). Expression sequences and distribution of two primary cell adhesion molecules during embryonic development of *Xenopus laevis*. *J. Cell. Biol.*, **105**, 2359.

MAGNANI, J.L. (1984). Carbohydrate differentiation and cancer associated antigens detected by monoclonal antibodies. *Biochem. Soc. Trans.*, **12**, 543.

MARCHALONIS, J.J., CONE, R.E. & SANTER, V. (1971). Enzymic iodination. A probe for accessible surface proteins in normal and neoplastic lymphocytes. *Biochem. J.*, **124**, 921.

McGARRY, R.C., HELFAND, S.L., QUARLES, R.H. & RODER, J.C. (1983). Recognition of myelin-associated glycoprotein by the monoclonal antibody HNK-1. *Nature*, **306**, 376.

MIRALDI, F., NELSON, A.D., ELLER, S. & 6 others (1986). Imaging of melanoma, osteogenic sarcoma and neuroblastoma using GD2 specific iodine 131 labelled monoclonal antibody. *J. Nucl. Med.*, **27**, 881.

MOORE, S.E., THOMPSON, J., KIRKNESS, V., DICKSON, J.G. & WALSH, F.S. (1987). Skeletal muscle neural cell adhesion molecule (NCAM): changes in protein and mRNA species during myogenesis of muscle cell lines. *J. Cell. Biol.*, **105**, 1377.

MORIKAWA, S., TATSUMI, E., BABA, M., HARADA, T. & YUSUHIRA, K. (1978). Two E-rosette forming lymphoid cell lines. *Int. J. Cancer*, **21**, 166.

PATEL, K., MOORE, S.E., DICKSON, G. & 4 others (1989*a*). Neural cell adhesion molecule (NCAM) is the antigen recognized by monoclonal antibodies of similar specificity in small cell lung carcinoma and neuroblastoma workshops. *Int. J. Cancer* (in the press).

PATEL, K., ROSSELL, R.J., MOORE, S.E., WALSH, F.S. & KEMSHEAD, J.T. (1989*b*). Monoclonal antibody UJ13A recognizes the neural cell adhesion molecule (NCAM) on human embryonic tumours. *Int. J. Cancer* (in the press).

SAITO, N., YU, R.K. & CHEUNG, N.K.V. (1985). Ganglioside GD2 specificity of monoclonal antibodies to human neuroblastoma cell. *Biochem. Biophys. Res. Commun.*, **127**, 1

SANTONI, M.J., BARTHELS, D., BARBAS, J.A. & 4 others (1987). Analysis of cDNA clones that code for the transmembrane forms of the mouse neural cell adhesion molecule (NCAM) and are generated by alternative RNA splicing. *Nucleic Acid Res.*, **15**, 8621.

SCHLESINGER, H.R., GERSON, J.M., MOORHEAD, P.S., MAGUIRE, H. & HUMMELER, K. (1976). Establishment and characterisation of human neuroblastoma cells lines. *Cancer Res.*, **36**, 3094.

SCHULTZ, G., CHERESH, D.A., VARKI, N.M., YU, A., STAFFILENO, L.K. & REISFELD, R.A. (1984). A murine monoclonal antibody detects ganglioside GD2 in tumour tissues and sera of neuroblastoma patients. *Cancer Res.*, **44**, 5914.

SCHWAB, M., ALITALO, K., KLEMPNAUERE, K.H. & 6 others (1983). Amplified DNA with limited homology to myc cellular oncogene is shared by human neuroblastoma cell lines and a neuroblastoma tumour. *Nature*, **305**, 245.

SEKIGUCHI, M., OOTA, T., SAKAKIBARA, K., INUI, N. & FUJII, G. (1979). Establishment and characterisation of a neuroblastoma cell line in tissue culture. *Jpn. J. Exp. Med.*, **49**, 7.

SHASHOUA, V.E., DANIEL, P.F., MOORE, M.E. & JUNGALWALA, F.B. (1986). Demonostration of glucuronic acid on brain glycoproteins which react with HNK-1 antibody. *Biochem. Biophys. Res. Commun.*, **138**, 902.

SMALL, S.J., SHULL, G.E., SANTONI, M.J. & AKESON, R. (1987). Identification of a cDNA clone that contains the complete coding sequence for a 145 kDa rat NCAM polypeptide. *J. Cell. Biol.*, **105**, 2335.

SUGIMOTO, T., TATSUMI, E., KEMSHEAD, J.T., HELSON, L., GREEN, A.A. & MINIWADA, J. (1984). Determination of cell surface membrane antigens common on both human neuroblastoma and leukaemic/lymphoma cell lines by a panel of 38 monoclonal antibodies. *J. Natl Cancer Inst.*, **73**, 51.

TUMILOWICZ, J.J., NICHOLS, W.W., CHOLON, J.J. & GREEN, A.E. (1970). Definition of a continuous human cell line derived from neuroblastoma. *Cancer Res.*, **30**, 2110.